# Novel Insights into the Cardioprotective Effects of Calcitriol in Myocardial Infarction

**DOI:** 10.3390/cells11101676

**Published:** 2022-05-18

**Authors:** Simin Yang, Chunmiao Wang, Chengshao Ruan, Meiling Chen, Ran Cao, Liang Sheng, Naiying Chang, Tong Xu, Peiwen Zhao, Xuesheng Liu, Fengqin Zhu, Qingzhong Xiao, Shan Gao

**Affiliations:** 1 Department of Pharmacology, Basic Medical College, Anhui Medical University, Hefei 230032, China; ysimin@163.com (S.Y.); ahmurcs@163.com (C.R.); chenml@cmpt.ac.cn (M.C.); caoran@ktz126.wecom (R.C.); sliang@cmpt.ac.cn (L.S.); changnaiying2022@163.com (N.C.); xt001928@163.com (T.X.); 13965869774@163.com (P.Z.); 2 Department of Cardiology, The First Affiliated Hospital, Anhui Medical University, Hefei 230022, China; wangchunmiao2004@163.com; 3 Cancer Hospital, Chinese Academy of Sciences, Hefei 230031, China; tczfq88@126.com; 4 Department of Anesthesiology, The First Affiliated Hospital, Anhui Medical University, Hefei 230022, China; liuxuesheng@ahmu.edu.cn; 5 Clinical Pharmacology, William Harvey Research Institute, Barts and The London School of Medicine and Dentistry, Queen Mary University of London, London EC1M 6BQ, UK

**Keywords:** calcitriol, vitamin D, myocardial infarction, cardiovascular disease, cardiac inflammation and remodeling, NF-κB, IL-10

## Abstract

Background: Increasing evidence indicates that vitamin D deficiency negatively affects the cardiovascular system. Here we studied the therapeutic effects of calcitriol in myocardial infarction (MI) and investigated its underlying mechanisms. Methods: A MI model of Kun-ming mice induced by left anterior descending coronary artery ligation was utilized to study the potential therapeutic effects of calcitriol on MI. AC16 human cardiomyocyte-like cells treated with TNF-α were used for exploring the mechanisms that underlie the cardioprotective effects of calcitriol. Results: We observed that calcitriol reversed adverse cardiovascular function and cardiac remodeling in post-MI mice. Mechanistically, calcitriol suppressed MI-induced cardiac inflammation, ameliorated cardiomyocyte death, and promoted cardiomyocyte proliferation. Specifically, calcitriol exerted these cellular effects by upregulating Vitamin D receptor (VDR). Increased VDR directly interacted with p65 and retained p65 in cytoplasm, thereby dampening NF-κB signaling and suppressing inflammation. Moreover, up-regulated VDR was translocated into nuclei where it directly bound to *IL-10* gene promoters to activate *IL-10* gene transcription, further inhibiting inflammation. Conclusion: We provide new insights into the cellular and molecular mechanisms underlying the cardioprotective effects of calcitriol, and we present comprehensive evidence to support the preventive and therapeutic effects of calcitriol on MI.

## 1. Introduction

Vitamin D is a type of fat-soluble molecule that is mainly responsible for promoting the absorption of phosphorus and calcium by intestinal mucosal cells [1]. It is historically known that vitamin D plays a pivotal role in bone and mineral metabolism. The vitamin D receptor (VDR) is ubiquitously expressed in most human tissues and organs, especially in blood vessels and the heart. It has been reported that individuals with vitamin D deficiency are more susceptible to a range of extraskeletal diseases. Additionally, increasing evidence supports a cardioprotective effect of vitamin D and the VDR pathway [2,3]. Nonetheless, the relationship between vitamin D levels and cardiovascular disease remains controversial. Edward et al. found that low-level 25-OH vitamin D3 was related to an elevated risk of MI [4]. In contrast, it was reported that vitamin D supplementation did not mitigate the incidence of invasive cancer or cardiovascular events [5]. The difference may be due to different clinical subjects, comorbidities, medications, as well as experimental conditions. Moreover, in clinical trials patients were administrated with the precursor of the vitamin D, and not with the active form, which may also account for the controversial findings in pre-clinical studies and clinical trials [6,7]. Therefore, whether or not the active form of vitamin D has cardioprotective properties remains unclear, and requires further investigations in both pre-clinical and clinical settings.

Calcitriol (1,25-dihydroxyvitamin D3), the active form of vitamin D3, is naturally made from vitamin D3 in the kidneys. Physiologically, calcitriol is responsible for maintaining calcium homeostasis in the intestines, bone, kidneys, and parathyroid glands. As a selective activator of the VDR, calcitriol is clinically used for the intervention of secondary hyperparathyroidism following chronic kidney disease [8]. However, the effects of calcitriol on preventing MI-induced adverse cardiac remodeling are unclear, and the precise mechanisms of action remain unexplored. In this study, using both preventive and therapeutic models, we demonstrate that calcitriol exhibits robust cardioprotective properties in both models, and this beneficial effect is attributed to its anti-inflammatory properties. More specifically, calcitriol binds and activates VDR signaling, which, in turn, inhibits the NF-κB signaling pathway but increases *IL-10* gene transcription and expression, thereby suppressing inflammatory response upon cardiac injury. Therefore, this study provides new insights into developing a novel strategic option for the treatment and intervention of MI.

## 2. Materials and Methods

### 2.1. Mice, MI Model, and Treatments

In total, 115 7-week-old male Kunming mice weighing 25–28 g were obtained from the Beijing Vital River Laboratory Animal Technology Corp. Ltd. (certificate No. SCXK (JING) 2016-0011) (Beijing, China) and maintained in the animal facility of Anhui Medical University. All mice were given free access to water and standard chow, housed in a room with a stable temperature (24–26 °C) and a 12-h light/dark cycle. These conditions are in line with the Guidelines for the Care and Use of Laboratory Animals as stated by the Ministry of Science and Technology of China, as well as in compliance with the welfare and ethics regulations of Anhui Medical University (No. 20,160,260, Hefei, China). MI was induced in the animals by a previously reported surgical method with only slight modifications [9]. In short, mice were anaesthetized intraperitoneally with pentobarbital sodium (60 mg/kg) injection. A 20-gauge polyethylene catheter was inserted into the trachea and a volume-cycled rodent respirator was set to generate 2–3 mL/cycle positive pressure ventilation and a respiratory rate of 120 cycles/min was maintained. The left anterior descending (LAD) artery was ligated with a 7-0 silk suture after thoracotomy at the level of the fourth rib and left sternal margin. The chest wall was closed with a continuous 6-0 prolene suture, followed by skin closure with a 4-0 polyester suture. We found that the death of experimental animals mainly occurred within the first three days after surgery, mainly due to ventricular rupture associated with inflammation or surgical complications, and all the remaining mice survived to the end of the experiment.

Sham surgery was also performed alongside MI induction procedures, which followed the same sequence of surgical maneuvers but without LAD ligation. The success of MI can be visually confirmed by the color changes to the left ventricular wall or the alterations in ECG. Calcitriol (Roche Pharmaceutical Co., Ltd., Shanghai, China) was solubilized in corn oil (Solarbio, Beijing, China, Cat# C7030). After LAD ligation, all mice were randomly divided into six groups and received the following drug administration via oral gavage once daily as described in previous studies [10,11]: (1) Sham group, 10 mL/kg/day corn oil (*n* = 9); (2) MI group, 10 mL/kg/day corn oil (*n* = 12); (3) Low dose calcitriol-treated group, 0.0375 μg/kg/day calcitriol (*n* = 12); (4) Medium dose calcitriol-treated group, 0.075 μg/kg/day calcitriol(*n* = 11); (5) High dose calcitriol-treated group, 0.15 μg/kg/day calcitriol (*n* = 12); (6) Fosinopril group, 2 mg/kg/day fosinopril (*n* = 11).

### 2.2. Triphenyltetrazolium Chloride (TTC) Staining and Infarct Size Measurement

Twenty-four hours after LAD ligation or at the end of each experimental protocol, mice were anesthetized with sodium pentobarbital (60 mg/kg, i.p.) injection and sacrificed. Blood was collected from the heart via cardiac puncture and the hearts were stored at −20 °C for 25 min for cryo-sectioning. The frozen heart tissues were cut into 2 mm thick short-axis sections, and the tissue slides were stained with 1% TTC solution (Biosharp, Hefei, China) for 20 min at 37 °C. Images were taken and the infarct area was quantified using Image J (1.61) (NIH, Bethesda, MD, USA).

### 2.3. Electrocardiogram (ECG)

Mice were anesthetized with pentobarbital sodium (60 mg/kg, i.p.) and placed onto a warm pad (at 37 °C) in a supine position. According to the ECG instructions, the four electrodes were connected to four limbs, and the ECG signals of all mice were recorded on the two leads of a four-channel direct-writing oscilloscope. In total, three recordings were taken for each animal (five minutes before surgery, five minutes after surgery, and four weeks after surgery), with each recording lasting for 60–120 s. The BL-420s biological data acquisition and analysis system (Chengdu Taimeng Technology Co., Ltd., Chengdu, China) was used for manual measurements which include: the height of P, QRS, J Point and T waves, the width of P waves, QRS, S and T waves, and the time of P-R and Q-T.

### 2.4. Echocardiographic Assessment

In order to assess the cardiac function of MI mice and the geometry of the infarcted area, echocardiography was performed 4 weeks after the LAD procedure, using an 18 MHz high-frequency scanning head (VINNO6, Suzhou VINNO Technology Co., Ltd., Suzhou, China) and left ventricular (LV) parasternal long-axis field M-mode imaging. The mean value of the data was based on measurements of at least six cardiac cycles, including left ventricular end systolic diameter (LVIDs), left ventricular end diastolic diameter (LVIDd), left ventricular end systolic anterior wall thickness (LVAWs), and left ventricular end diastolic anterior wall thickness (LVAWd). The ejection fraction (EF) and fractional shortening (FS) were calculated by the computer’s own software (VINNO6, Suzhou VINNO Technology Co., Ltd., Suzhou, China).

### 2.5. Sample Collection and Histology

At the end of the study protocols, 1.5 mL of blood samples were rapidly collected and subsequently centrifuged at 4000 r/min for 12 min at 4 °C. The supernatant was stored at −80 °C for further study. After weighing both hearts and lungs, the heart weight to body weight (HW/BW) ratio and lung weight to body weight (LUW/BW) ratio were calculated. After being photographed, the heart samples were fixed in 10% neutral buffered formalin solution for histological analysis or directly stored at −80 °C for future Western blotting study. In addition, thoracic aortas were isolated from mice and preserved in a 10% neutral buffered formalin solution for histological analyses. Both hearts and aortas were embedded into paraffin blocks and cut into 5 µm paraffin sections, followed by Hematoxylin-eosin staining (HE staining), Masson staining (Solarbio), and other histopathological analysis as detailed below.

Cardiac tissues were fixed with 10% neutral formalin, dehydrated, embedded in paraffin, and sectioned with a Leica tissue microtome. For Masson-trichrome staining, tissue sections were incubated at 60 °C for 2 h and deparaffinized, followed by incubation with Bouin (A) solution at 37 °C for 2 h. The sections were rinsed with running water until the yellow color disappeared and then dipped into Lazuli blue solution for 2–3 min. After being rinsed lightly with water, sections were dipped into Mayer’s hematoxylin solution for 2–3 min and differentiated with acidic ethanol differentiation solution for a few seconds. After being rinsed with running water for 10 min, sections were incubated with Ponceau red-magenta solution for 10 min, followed by Phosphomolybdic acid treatment for another 10 min. After removing the staining solution, sections were stained with aniline blue staining solution for 5 min, treated with a weak acid solution for 2 min, and dehydrated quickly with 95% and absolute ethanol three times. Finally, sections were cleared with xylene three times, dried in an incubator, and sealed with neutral resin. Sections were inspected under the microscope, and images were taken. The volume fraction of interstitial collagen in the whole Masson-stained heart was measured by an automatic image analyzer (Image-Pro Plus 3.0, Media Cybernetics, Silver Spring, MD, USA). Image J 1.61 software was used to analyze and calculate the myocardial collagen volume fraction (CVF) and perivascular collagen area (PVCA), respectively.

### 2.6. Aortic Tension Measurement

Isolated thoracic aortas were prepared as described in a previous study [12]. The transverse ring was sheared and mounted onto the DMT 620M vascular tension measurement system (Danish Myo Technology A/S, Aarhus, Denmark). Each chamber was filled with physiological salt solution (PSS) (unit, mM/L: KH_2_PO_4_, 1.18; KCI, 4.7; glucose, 5.5; NaCl, 130; CaCl_2_, 1.16; MgSO_4_, 1.17; EDTA, 0.026; NaHCO_3_, 14.9), aerated with a gas mixture consisting of 5% CO_2_ and 95% O_2_. The initial aortic tension was incrementally adjusted to 6 mN and allowed to stabilize for 1 h (fresh PSS medium was added 2–3 times after the initial strain was established). Next, KPSS solution (unit, mM/L: CaCl_2_, 1.6; NaCl, 74.7; NaHCO_3_, 14.9; KCI, 60; KH_2_PO_4_, 1.18; glucose, 5.5; MgSO_4_, 1.17; EDTA, 0.026) was added to depolarize the vessel for 15 min, and the chamber was then washed with fresh PSS solution every 10 min for a total of 3 times. This process of adding KPSS and washing with PSS was repeated two more times. After being equilibrated to the standard initial strain (6 mN), phenylephrine (PE) (10^−5^ M/L) was added to induce vasoconstriction. After brief stabilization, various concentrations of acetylcholine (Ach) (10^−9^~10^−4^ M/L) or sodium nitroprusside (SNP) (10^−9^~10^−4^ M/L) were added to induce vasodilation. SNP was used for excluding other factors that might affect vasodilatation.

### 2.7. Enzyme-Linked Immunosorbent Assay

Serum levels of NT-proBNP (Cat# JL11641 Jianglaibio, Shanghai, China), cTnI (Cat# JL46089 Jianglaibio, Shanghai, China), 25-OH vitamin D3 (Cat# JL20117 Jianglaibio, Shanghai, China), TNF-α (Cat# JL10484 Jianglaibio, Shanghai, China), IL-10 (Cat# JL20242 Jianglaibio, Shanghai, China), and IL-1β (Cat# JL18442 Jianglaibio, Shanghai, China) were measured by mouse enzyme-linked immunosorbent assay (ELISA) kits according to the manufacturer’s instructions.

### 2.8. Protein Extraction and Western Blotting

Fresh left ventricular cardiac tissues and AC16 cells were pulverized for protein extraction. Briefly, 50 mg of tissue samples were mixed with 500 μL RIPA lysis buffer (containing 5 μL protease inhibitor and 5 μL phosphatase inhibitor) and ground twice with a tissue grinder for 60 s at 4 °C. For AC16 cells, cells were lysed with 200 μL RIPA lysis buffer (containing 2 μL protease inhibitor and 2 μL phosphatase inhibitor), scraped, and collected into an EP tube. After being incubated on ice for 30 min, samples were centrifuged at 12,000 r/min at 4 °C for 15 min, and the supernatant was collected for analysis.

A Nuclear Protein Extraction Kit (Solarbio, R0050) was used to extract nuclear proteins including VDR according to the manufacturer’s instructions. Briefly, 50 mg tissue samples were cut into very small pieces in 500 μL PBS, followed by homogenization on ice with a tissue homogenizer. After homogenization, tissue lysates were centrifuged at 500 g for 2–3 min at 4 °C. The supernatant was discarded and the pellet was collected. After adding 200 μL of plasma protein extraction reagent, samples were vortexed at high speed for 15 s and incubated on ice for 10 min. After vigorously vortexing the samples for 10 s at the highest speed, samples were centrifuged at 12,000 g for 10 min at 4 °C. The supernatant was collected and used as the cytoplasmic protein, while the pellet was dissolved in 50–100 μL nuclear protein extraction reagent and used as nuclear fractionation.

Once extracted, protein concentration was quantified by a bicinchoninic acid assay (BCA) kit (Beyotime Biotechnology, Shanghai, China) and equalized to the same concentration. Total protein samples from each group were separated in 8–15% SDS-PAGE and transferred onto polyvinylidene fluoride (PVDF) membranes. The membranes were blocked with 5% nonfat milk in TBST for 1.5 h, followed by incubation in a primary antibody solution at 4 °C overnight: anti-TNF-α (Affinity Cat# AF7014 RRID: AB_2835319), anti-IL-1β (Affinity Cat# AF5103 RRID: AB_2837589), anti-IL-10 (Affinity Cat# DF6894 RRID: AB_2838853), anti-NF-κB p65 (Abcam Cat# ab16502 RRID: AB_443394), anti-P-NF-κB p65 (WanleiBio Cat# wl02169), anti-VDR (Affinity Cat# AF6159 RRID: AB_2835028), histone H3 (Affinity Cat# AF0863 RRID: AB_2810277), PCNA (Affinity Cat# AF0239 RRID: AB_2833414), β-actin (Beijing Zhongshan Golden Bridge Biotechnology, Beijing, China; Cat# TA-09), and GAPDH (Affinity Cat# AF7021 RRID: AB_2839421). After being washed with TBST, the membranes were incubated with goat anti-rabbit IgG obtained from Affinity (Cat# S0001 RRID: AB_2839429) and Beijing Zhongshan Golden Bridge Biotechnology Co., Ltd. (Cat# ZB-2305) for one hour at room temperature. A chemiluminescence kit (ECL; Amersham Biosciences, Little Chalfont, UK) was used to allow detection of target protein expression; the blot was then imaged and analyzed by Image J 1.61 software for quantification of band intensity.

### 2.9. Quantitative Real-Time Polymerase Chain Reaction (qRT-PCR)

Total RNAs were extracted from cells using the MagMAX total RNA isolation kit (Thermo Fisher Scientific, Waltham, MA, USA) and reverse-transcribed into cDNAs using Prime Script (Takara, Dalian, China). Using respective primers (Supplementary Table S1), qRT-PCR was performed in duplicate using a CFX96 Real-Time PCR Detection System. Expression levels were normalized to the expression of β-actin mRNA. The relative expression was calculated by the 2(^–ΔΔCt^) method.

### 2.10. Cell Culture and Treatment

AC16 cells purchased from Beinna Biotech (BNCC337712; Beijing, China) were maintained in DMEM (F12, HyClone (SH30023.01), Logan, UT, USA) supplemented with 10% fetal bovine serum (FBS; Hyclone), 100 U/mL penicillin, and 100 μg/mL streptomycin in a chamber of 5% CO_2_ at 37 °C. Upon reaching 70–80% confluency, calcitriol (dissolved in DMSO) was added to the medium. In some experiments, cells were incubated with different concentrations of TNF-α (5, 10, 15, 20, 25 ng/mL, diluted with sterilized deionized water) or with 20 ng/mL TNF-α for 3 h, 6 h, 12 h, 24 h, and 48 h, respectively, as described in previous studies [13,14]. Four groups were included in other experiments: (1) control group; (2) TNF-α group: the AC16 cells were stimulated with TNF-α (20 ng/mL) for 24 h; (3) TNF-α plus Calcitriol-L group: the AC16 cells were simultaneously given TNF-α (20 ng/mL) and calcitriol (10 nmol/L) for 24 h; or Calcitriol-H group: calcitriol (100 nmol/L) for 24 h; (4) TNF-α plus Calcitriol-H group: the AC16 were simultaneously treated with TNF-α (20 ng/mL) and calcitriol (100 nmol/L) for 24 h.

### 2.11. TUNEL Assay

DNA fragmentation was detected by the TUNEL apoptosis assay kit (C1090, Beyotime Biotechnology, Shanghai, China). All the nuclei were stained blue with DAPI, whereas the nuclei for apoptotic or dead cells were stained red. For quantification, apoptotic cells were calculated by counting the number of positive nuclei (red fluorescence) per 100 nuclei (blue fluorescence) and taking the average from three different fields-of-view.

### 2.12. Cell Viability Assay

Cell viability was measured by a Cell Counting Kit-8 (CCK-8) assay kit (IV08-500T, Invigentech, Irvine, CA, USA) according to the manufacturer’s guidelines. Cells were seeded in 96-well plates at a density of 5 × 10^3^ cells/well prior to cell viability assay. During the measurement, 10 μL of CCK-8 reagent was added to each well, and the plate was cultured for an additional 1–4 h at 37 °C. The absorbance of each well was measured at 450 nm using a microplate reader (Bio-Tek Company, Winooski, VT, USA).

### 2.13. Immunofluorescence Analysis

Myocardial VDR proteins were detected by immunofluorescence staining in cardiac tissue paraffin sections. Slides were deparaffinized, rehydrated, and then boiled in a microwave for at least 10 min for antigen retrieval. After being blocked in block solution for 30 min, slides were incubated with primary rabbit anti-VDR antibody or rabbit IgG at 4 °C overnight, then with a secondary goat anti-rabbit antibody (1:50; 713-076-147; Jackson Immuno Research Laboratories, Inc., WestGrove, PA, USA) for 1 h at 37 °C. All sample tissues were stained with 4′6-diamidino-2-phenylindole (DAPI) prior to imaging. For cell staining*,* AC16 cells were plated on the coverslips and incubated for 24 h. After being subjected to the indicated treatment, the cells were fixed in paraformaldehyde and permeabilized in 10% Triton in PBS for 5 min, then blocked in 3% BSA for 1 h, followed by overnight incubation with respective primary antibodies as indicated in each figure at 4 °C. After washing, the coverslips were incubated with a secondary antibody at 37 °C for 1 h and then stained with DAPI. Confocal images of myocardial cells and AC16 cells were captured with a ZEISS LSM880 + airyscan confocal microscope (Jena, Germany).

### 2.14. Imaging Flow Cytometry

AC16 cells were stained with anti-NF-κB/p65-FITC, followed by DAPI staining. In total, 10,000 events were collected on the Amnis ImageStreamX Mark II (ISX MKII) Imaging Flow Cytometer (Amnis, Merck Millipore, Seattle, WA, USA) with 488 nm laser excitation. Cells were graded and gated in order to quantify the single-cell population that was positive for DAPI and p65, and 75–85% of the collected images were included in the final analysis. After data collection, the “similarity” feature in the IDEAS^®^ (Image Data Exploration and Analysis) software package was used to measure the spatial relationship between NF-κB/p65 and DAPI staining. “Similarity score” (SS) is the Pearson correlation coefficient of logarithmic transformation of pixel values for p65 and DAPI, which provides a value for the degree of nuclear localization of NF-κB/p65. No correlation between the images of cells with a low similarity score (corresponding to the forward cytoplasmic distribution of NF-κB/p65) was observed, while a positive correlation between the images of cells with a high SS score (corresponding to the main nuclear distribution of NF-κB/p65) was observed.

### 2.15. Measurement of 1,25(OH)_2_-VitD_3_ Contents in AC16 Cells

The nuclear and cytoplasmic lipid contents were isolated with an extraction kit (310001, BestBio, Shanghai, China), and the levels of 1,25(OH)_2_-VitD_3_ in the nucleus or cytoplasm were analyzed by LC-MS/MS (Hangzhou Kailai Spectrum Medical Laboratory Co., Ltd., Hangzhou, China).

### 2.16. Co-Immunoprecipitation (Co-IP) Assay

AC16 cells with the indicated treatments were collected and homogenized in lysis buffer (abs955, Absin, Shanghai, China) on ice for at least 5 min. After centrifugation at 14,000 g for 10 min, the cell lysates (500 μL) were incubated with 5 μL of protein agarose A/G beads (abs955, Absin, Shanghai, China) at 4 °C for 30–60 min to reduce non-specific bindings. The supernatant was collected and incubated with 2.0 μg rabbit anti-VDR antibody (Affinity Cat# AF6159 RRID: AB_2835028) overnight on a rotating plate; 5 μL agarose beads (Cat#abs955, Absin, Shanghai, China) were then added to each sample and incubated at 4 °C for 1–3 h. Lastly, the precipitate was washed three times with washing buffer (abs955, Absin, Shanghai, China) and subjected to Western blot analysis.

### 2.17. VDR siRNA Transfection

*VDR* siRNA (GenePharma, Shanghai, China) was transiently introduced into AC16 cells at 50% confluency using LipofectamineTM 3000 (Invitrogen, Carlsbad, CA, USA). After 48 h of transfection, the medium was changed to a standard DMEM medium, and cells were induced with TNF-α (20 ng/mL) for 24 h. Scramble siRNA was used as a negative control. Gene silencing was validated by qRT-PCR and Western blot with anti-VDR antibody (Affinity Cat# AF6159 RRID: AB_2835028), respectively.

### 2.18. Plasmid Construction and Luciferase Reporter Assay

The DNA sequence containing wild-type (WT) and mutated RXRA::VDR binding element and its respective up- and down-stream sequence (300 bps in total) of human *IL-10* gene promoter were synthesized, and sub-cloned into the pGL3-basic vector (Promega, Cat# E1751), and designated as pGL3-hu-IL10-WT and pGL3-hu-IL10-Mut, respectively. The resultant vectors were verified by DNA sequencing.

AC16 cells were co-transfected with respective gene promoter vectors and control or *VDR* gene-specific siRNAs, followed by various treatments as indicated in the figures. pTK-Renilla (20 ng per well; Promega, Cat# E2241) was included in all transfection assays as an internal control. Dual-luciferase activity assays were conducted 48 h after transfection using a standard protocol. The relative luciferase unit (RLU) was defined as the ratio of luciferase versus renilla activity to that of the control (set as 1.0).

### 2.19. ChIP-qPCR Assay

The ChIP assays were performed using the EZ CHIP KIT (Millipore, Cat# 17-371). Briefly, AC16 cells were harvested and sonicated, followed by immunoprecipitation with 5 µg VDR antibody or normal rabbit IgG. The immunoprecipitates were eluted from the beads using 100 μL elution buffer (50 mM NaHCO3, 1% SDS) and deproteinized using proteinase K. Immunoprecipitated DNA was extracted, purified, and then used to amplify target DNA sequences by qRT-PCR. Promoter DNA enrichment with a specific antibody was calculated using the percent input method.

### 2.20. Statistical Analysis

In imaging flow cytometry, statistical analysis was carried out by IDEAS software (Amnis-ImageStream Imaging Flow Cytometer) and one-way ANOVA with the Tukey–Kramer exact probability test. Other statistical analyses were performed using GraphPad Prism 9 (GraphPad Software, San Diego, CA, USA) and considered significant if *p* < 0.05 as detailed in each figure.

## 3. Results

### 3.1. Calcitriol Improves Cardiac Function and Ameliorates Adverse Cardiac Remodeling in Post-MI Mice

**Figure 1:**
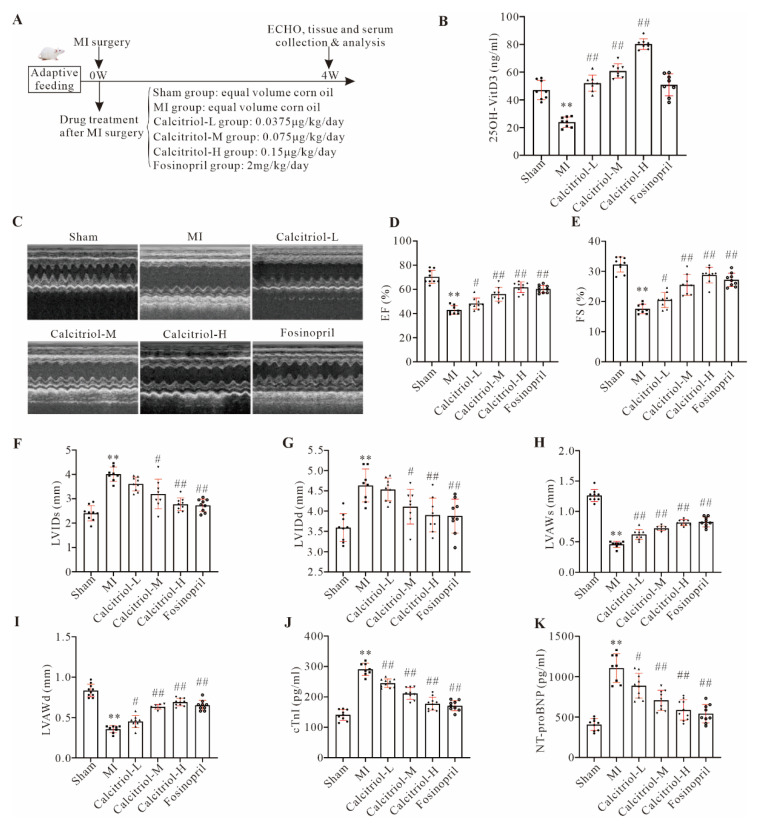
Calcitriol improved cardiac function in post-MI mice. (**A**) Experimental protocol of the in vivo study with the indicated treatments. All the samples/data were collected at the end of the study protocol (4 weeks post-MI/treatments). (**B**) Serum 25-OH vitamin D3 in mice with the indicated treatments. (**C**–**I**) Cardiac function analysis by echocardiogram. Representative images of echocardiography with M-mode (**C**) and quantitative analysis of EF (**D**), FS (**E**), LVIDs (**F**), LVIDd (**G**), LVAWs (**H**), and LVAWd (**I**) from eight or more mice, were presented here. (**J**,**K**) Serum cTnI and NT-proBNP levels in mice. (Data represent means ± S.D. *n* ≥ 8, * *p* < 0.05, ** *p* < 0.01 versus sham group, ^#^ *p* < 0.05, ^##^ *p* < 0.01 versus MI group; one-way ANOVA with a post hoc test of Tukey’s analysis).

In order to investigate the possible effects of calcitriol on cardiac functions and remodeling after MI, mice with or without MI were daily administered with different concentrations of calcitriol or fosinopril for four weeks as shown in Figure 1A. As expected, serum 25-OH vitamin D3 level was significantly decreased in MI mice at 4 weeks post-surgery, compared to the sham control (Figure 1B). Serum 25-OH vitamin D3 levels in MI mice were increased by calcitriol in a dose-dependent manner, but not by fosinopril (Figure 1B), confirming successful vitamin D3 administration. Moreover, an ECG was conducted before and immediately after LAD ligation to confirm the success of the MI model (Figure S1A,B), which was further confirmed by TTC staining. TTC staining showed that the ligation-induced infarction area was approximately 39.81 ± 3.21% at 24 h post-MI (Figure S1C). Echocardiography data showed that both calcitriol and fosinopril could significantly improve cardiac functions as evidenced by increased EF and FS in MI mice treated with calcitriol or fosinopril (Figure 1C–E). Moreover, compared to the sham group, mice in the MI group displayed a significant increase in both LVIDs and LVIDd but a dramatic decrease in LVAWs and LVAWd, suggesting that MI causes ischemic cardiomyopathy. Importantly, MI-induced ischemic cardiomyopathy was reversed by both calcitriol and fosinopril (Figure 1F–I). Furthermore, ECG analysis revealed that all the MI-induced cardiac functional abnormalities were completely or partially remedied by calcitriol or fosinopril at four weeks post-MI (Figure S1A,B). Finally, serum levels of cTnI and NT-proBNP were markedly increased in MI mice compared to the sham operation group, which were attenuated in treatment groups of calcitriol and fosinopril (Figure 1J–K). Importantly, we observed that calcitriol restored cardiac functions and structure in a dose-dependent manner (Figure 1C–K).

### 3.2. Calcitriol Reverses MI-Induced Adverse Cardiac Remodeling

**Figure 2:**
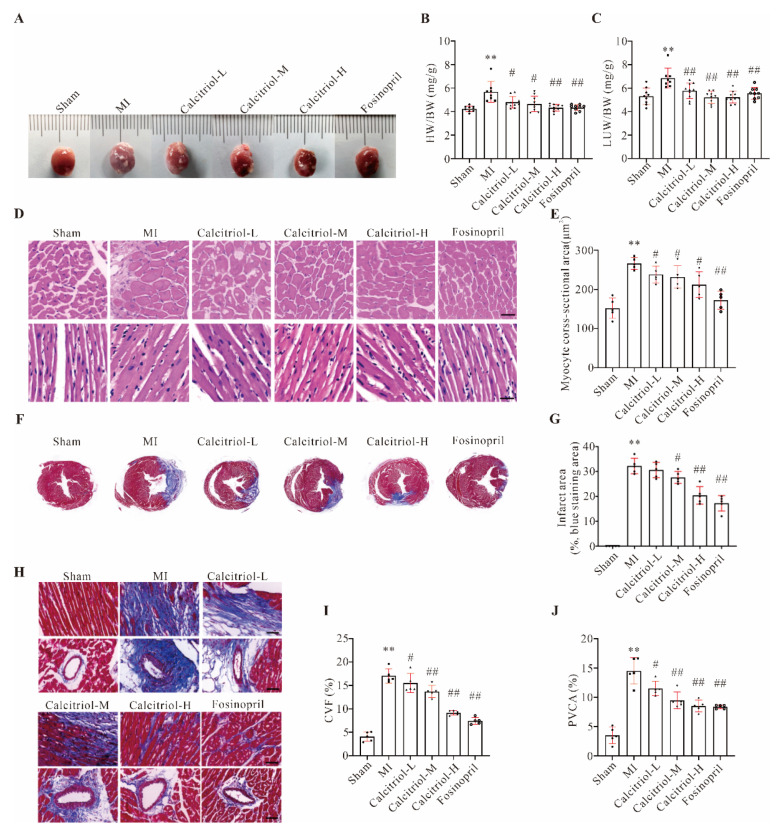
Calcitriol reversed MI-induced adverse cardiac remodeling. (**A**) Representative macroscopic heart images. (**B**,**C**) Ratios of heart (HW, **B**) or lung (LUW, **C**) weight to body weight (BW) (mg/g) (*n* ≥ 8). (**D**,**E**) H&E staining analysis of the cross-sections of the middle part of murine hearts. Representative images (**D**) and quantitative analysis of myocyte cross-sectional area (CSA, **E**) from five mice (*n* = 5 mice for each group) were presented here. Scale bar represents 50 μm. (**F**,**G**) Masson-trichrome staining analysis of the cross-sections of the middle part of murine hearts. Representative images (**F**) and quantitative analysis of infarct area (%, blue staining area, **G**) from five mice (*n* = 5 for each group) were presented here. (**H**–**J**) Cardiac fibrosis analysis of the cross-sections of the middle part of murine hearts. Representative images of myocardial and perivascular fibrosis (**H**), and quantitative analysis of collagen volume fraction (CVF, %, **I**) and perivascular collagen area (PVCA, %, **J**) from five mice were presented here (*n* = 5 mice for each group). Three images from each mouse were measured and averaged in quantitative analysis. (Scale bar represents 50 μm. Data represent means ± S.D. ** *p* < 0.01 versus sham group, ^#^ *p* < 0.05, ^##^ *p* < 0.01 versus MI group; one-way ANOVA with a post hoc test of Tukey’s analysis).

As expected, compared to the sham group, both heart volumes and heart weight (HW)/body weight (BW) index, as well as lung weight (LUW)/body weight (BW) index, were significantly increased in the MI group, and such increases were attenuated in all treatment groups at 4 weeks (Figure 2A–C). HE staining data further supported these macroscopic observations, as evidenced by the cross-sectional area (CSA) of cardiomyocytes in MI mice being much larger than that of sham mice, while calcitriol treatment, especially with a high-dose (0.15 μg/kg/day), significantly reduced it (Figure 2D–E). Meanwhile, Masson staining demonstrated that the MI-induced infarct area was dramatically reduced by calcitriol and fosinopril (Figure 2F–G). Moreover, MI-induced cardiac fibrosis was significantly attenuated by calcitriol and fosinopril, as evidenced by both myocardial collagen volume fraction (CVF) and perivascular collagen area (PVCA) in MI mice being remarkably diminished by drug interventions (Figure 2H–J). These data have collectively demonstrated that calcitriol could attenuate MI-induced pathological remodeling.

### 3.3. Calcitriol Treatment in Established Chronic MI Halt Disease Progression

**Figure 3:**
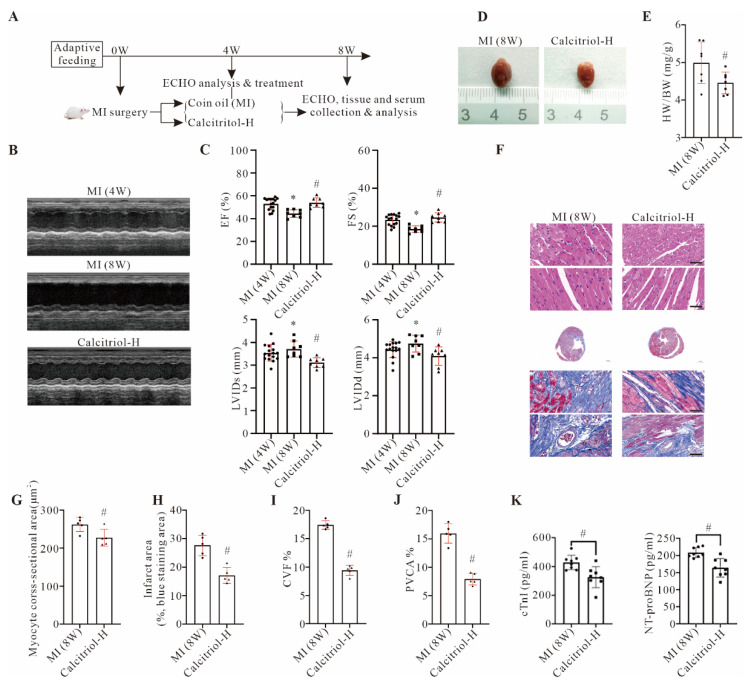
Calcitriol treatment in established chronic MI halted disease progression. (**A**) Experimental protocol of the therapeutic study. From week 5, mice in the MI group were further randomly divided into two treatment groups and received corn oil (MI(8W)) and high dose calcitriol (Calcitriol-H), respectively. (**B**,**C**) Cardiac function analysis by echocardiogram. (**B**) Representative echocardiography images of mice with MI surgery (MI (4W)) or MI surgery with (Calcitriol-H)) or without (MI (8W)). Calcitriol-H treatment starting from five weeks post MI. (**C**) Quantitative analysis of EF, FS, LVIDs, and LVIDd from seven mice with different treatments (*n* = 7). Representative macroscopic heart images (**D**) and the ratio of HW/BW (mg/g) (**E**) were shown here. (**F**–**J**) HE and Masson-trichrome staining analysis. Representative images (**F**) and quantitative analysis of myocyte CSA (**G**), infarct area (**H**), CVF (**I**), and PVCA (**J**) from five mice (*n* = 5) were presented here. (**K**) Serum NT-proBNP and cTnI levels in mice with the indicated treatment (*n* = 8) (Scale bar represents 50 μm. Data represent means ± S.D. * *p* < 0.05 versus MI (4W) group, ^#^ *p* < 0.05 versus MI (8W) group. Unpaired student *t*-test was used in (**E**,**G**–**K**), while one-way ANOVA with a post hoc test of Tukey’s analysis was used in **C**).

Our data so far demonstrated that calcitriol treatment, particularly with a high dose, could restore cardiac functions and structural impairments induced by MI in post-MI mice. To further explore the therapeutic value of calcitriol in established MI, a therapeutic interventional protocol as shown in Figure 3A was used in this study. As expected, the echocardiography (Figure 3B,C) data showed that while the mice in the control group (MI (8W)) exhibited continuous deterioration of cardiac functions, the high dose of calcitriol treatment starting from week 5 could stop or even reverse such cardiac functional and structural deterioration, and halt disease progression. Moreover, calcitriol treatment starting from week 5 post-MI also significantly reduced cardiac pathological remodeling and fibrosis, as demonstrated in HE and Masson staining analysis (Figure 3F–J). A similar effect was observed in serum cTnI and NT-proBNP (Figure 3K). These data have collectively confirmed the therapeutic potential of calcitriol in established chronic MI.

### 3.4. Calcitriol Reverses MI-Induced Adverse Aortic Remodeling and Restores Vascular Reactivity

HE staining showed that MI induced pathological aortic remodeling, as evidenced by the increased area of total aortic wall area (TAA), luminal radius (L), aorta radius (AR), and media thickness (M) in MI mice compared with sham ones (Figure S2A–E). Importantly, this MI-induced pathological remodeling was reversed by calcitriol and fosinopril (Figure S2A–E). Meanwhile, Masson staining showed that calcitriol or fosinopril attenuated MI-induced aortic collagen deposition (Figure S2F–G). As impaired endothelial-dependent vasodilation is one of the main contributors to cardiac injury post-MI, vascular tension was assessed using a DMT 620M vascular tension measurement system. We found that MI impaired acetylcholine (Ach)-mediated vasodilatation, and such impairment was blocked by calcitriol and fosinopril (Figure S2H). Interestingly, high-dose calcitriol could completely restore vascular dysfunction induced by MI (Figure S2H). However, no similar effect was observed in sodium nitroprusside (SNP)-mediated vasodilatation (Figure S2I). Taken together, these results suggest that reversing pathological aortic remodeling and restoring vascular reactivity are two beneficial effects of calcitriol on MI, among its other cardioprotective properties.

### 3.5. Calcitriol Inhibits the Cardiac Apoptosis and Inflammation by Modulating VDR Signaling

**Figure 4:**
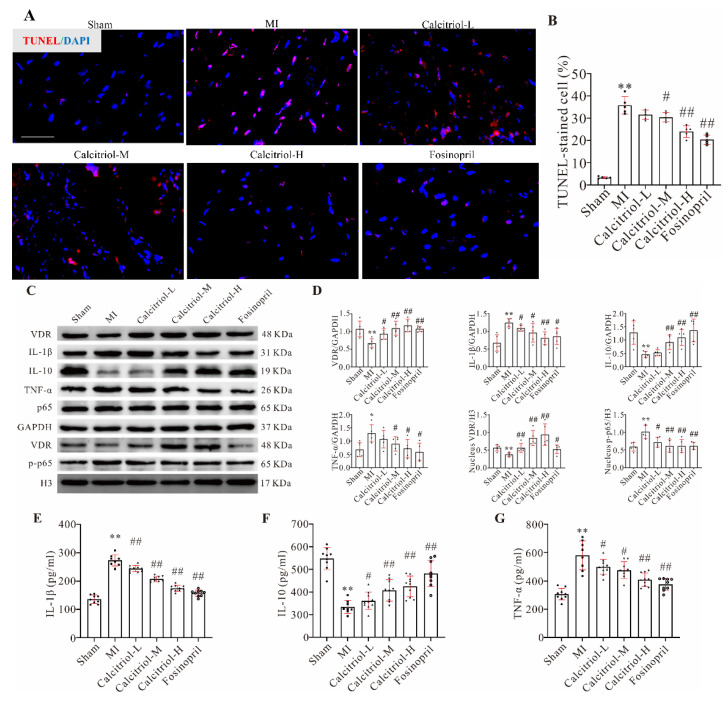
Calcitriol inhibited MI-induced cardiac myocyte apoptosis and death, as well as inflammation by modulating VDR signaling. (**A**,**B**) Calcitriol decreased myocyte apoptosis and death. Representative photographs (**A**) and quantitative analysis of TUNEL staining of the cardiomyocytes in mice with the indicated treatments (*n* = 5). Scale bar represents 50 μm. (**C**,**D**) Western blot analysis of protein expressions in the whole-cell lysates (VDR, IL-1β, IL-10, TNF-α, and NF-κB p65) or nuclear lysate (VDR and p-NF-κB p65) in mice with various treatments (*n* = 5). (**E**–**G**) Serum IL-1β, TNF-α, and IL-10 levels in mice with the indicated treatments (*n* ≥ 8). Data presented here are representative images (**A**,**C**) or quantitative analysis (**B**,**D**–**G**), respectively. (Scale bar represents 50 μm. Data represent means ± S.D. * *p* < 0.05, ** *p* < 0.01 versus sham group, ^#^ *p* < 0.05, ^##^ *p* < 0.01 versus MI group; one-way ANOVA with a post hoc test of Tukey’s analysis).

Cardiomyocyte apoptosis is a key determinant of MI-induced cardiac remodeling. TUNEL assay showed that MI induced a large number of cell apoptosis or death, which was significantly reduced by medium and high doses of calcitriol (Figure 4A,B). Vitamin D exerts its cellular functions mainly through the vitamin D receptor (VDR). Indeed, immunofluorescence analysis showed that calcitriol, but not fosinopril, could increase cardiac VDR expression in MI mice (Figure S3), which was further confirmed by Western blot analysis (Figure 4C,D). Interestingly, both total and nuclear VDR expression levels were upregulated by calcitriol in a dose-dependent manner (Figure 4C,D).

The NF-κB signaling pathway plays a predominant role in inflammatory cytokines activation, causing tissue inflammation and cell apoptosis [15]. Thus, we hypothesized that calcitriol exerts its cardioprotective effect by modulating the NF-κB signaling pathway. Data shown in Figure 4C,D revealed that while the total p65 expression level was changed by none of these treatments, MI-induced NF-κB p-p65 nuclear translocation was blunted by both calcitriol and fosinopril. Consequently, MI-induced expressions of the pro-inflammatory cytokines IL-1β and TNF-α in cardiac tissues were significantly reduced by these two drugs, while an opposite effect was observed in IL-10 protein expression, an anti-inflammatory cytokine (Figure 4C,D). Similar trends were observed with serum IL-1β, TNF-α, and IL-10 (Figure 4E–G). These data have collectively shown that calcitriol reduces cardiac and systemic inflammation, thereby exerting its cardioprotective effect in post-MI mice.

### 3.6. Calcitriol Rescues Cardiomyocytes from TNF-α-Induced Death, Reduces Inflammation, and Promotes Cardiomyocyte Proliferation

**Figure 5:**
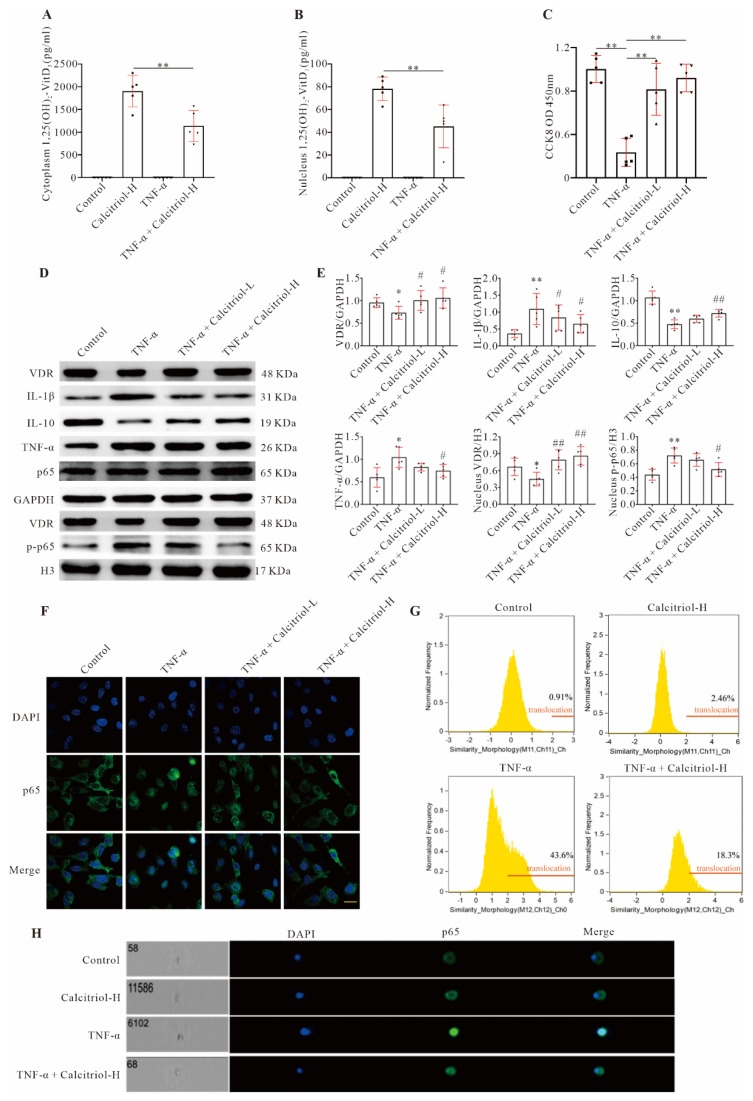
Calcitriol inhibited TNF-α-induced cardiomyocyte inflammation by preventing P65 nuclear translocation. (**A**,**B**) The cytoplasm (**A**) and nuclear (**B**) 1,25-OH Vitamin D_3_ levels in TNF-α-treated AC16 cells (*n* = 5). (**C**) CCK8 assay showed cell viability in AC16 cells exposed to 20 ng/mL TNF-α for 24 h in the absence or presence of different concentrations of calcitriol (*n* = 5). (**D**,**E**) Western blot analysis of protein expressions in the whole-cell lysates (VDR, IL-1β, IL-10, TNF-α, and NF-κB p65) or nuclear lysate (VDR and p-NF-κB p65) in AC16 cells with various treatments. Representative images (**D**) or quantitative analysis (**E**) from five experiments (*n* = 5) were presented here. (**F**) Representative immunofluorescence staining analysis of p65 expression in AC16 cells. Scale bar represents 50 μm. (**G**,**H**) Imaging Flow Cytometer analysis of NF-κB p65 cellular locations in AC16 cells with the indicated treatments. Representative images (**H**) and quantitative data of NF-κB p65 nuclear translocation assessed by IDEAS^®^ Software were presented here, respectively. (Scale bar represents 50 μm. Data represent means ± S.D. * *p* < 0.05, ** *p* < 0.01 versus sham group, ^#^ *p* < 0.05, ^##^ *p* < 0.01 versus TNF-α; one-way ANOVA with a post hoc test of Tukey’s analysis).

As expected, TNF-α reduced AC16 (a human cardiomyocyte cell line) cell viability in a dose- and time-dependent manner (Figure S4A,B). Although high levels of 1,25(OH)2-VitD3 could be detected in both the cytoplasm and nuclei of AC16 cells treated with a low (10 nM) or high (100 nM) concentration of calcitriol (Figure 5A,B), AC16 cell viability was not affected by calcitriol treatment alone (Figure S4C). However, TNF-α-induced decreased cell viability was almost rescued by a high concentration of calcitriol (Figure 5C). Moreover, we found that both the cell numbers and percentage of confluency of cultured AC16 cells were gradually decreased upon TNF-α treatment, which was partially reversed by calcitriol (Figure S4D,E). Furthermore, Western blot and immunofluorescence results showed that TNF-α significantly decreased AC16 cell proliferation, as evidenced by decreased expression levels of PCNA (Figure S4F,G) and lower percentages of PCNA-/Ki67-positive cells (Figure S4H), respectively. Such reductions were partially reversed by high-dose calcitriol, suggesting a promotive role for calcitriol in cardiomyocyte proliferation or cardiac regeneration. Finally, immunofluorescence staining with antibody against cleaved caspase-3 showed that while TNF-α treatment induced a high level of cleaved caspase-3 expression compared to control treatment, calcitriol could blunt such an induction (Figure S4I,J), further confirming an inhibitory effect of calcitriol on cardiomyocyte apoptosis. Similar to in vivo observations, the addition of calcitriol to TNF-α-treated AC16 cells could reduce IL-1β and TNF-α, but increase IL-10 expression (Figure 5D,E). We also observed that calcitriol treatment could increase VDR expression in both cytoplasmic and nuclear compartments (Figure 5D,E, Figure S5). Finally, we observed that TNF-α-induced p65 nuclear translocation was blunted by calcitriol treatment, as demonstrated by Western blot (Figure 5D,E), immunofluorescence (Figure 5F), and Imaging Flow Cytometer analysis (Figure 5G,H). These data have collectively shown that, consistent with in vivo findings, preventing TNF-α-induced NF-κB p-p65 nuclear translocation underpins the anti-inflammatory and cardioprotective effects of calcitriol in the context of MI.

### 3.7. VDR Is Essential for the Anti-Inflammatory Effect of Calcitriol in TNF-α-Treated AC16 Cells

**Figure 6:**
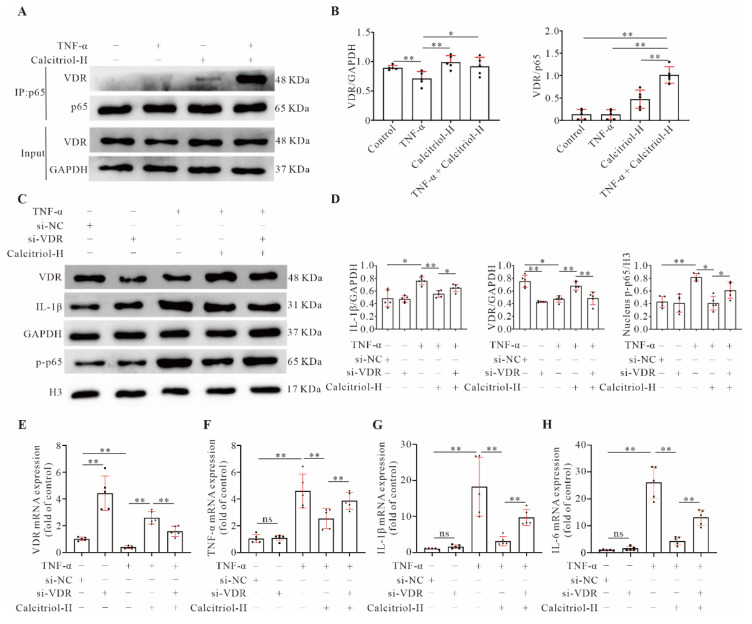
Importance of VDR in calcitriol-regulated inflammatory cytokines expressions. (**A**,**B**) Calcitriol increased the interaction of VDR with p65. AC16 cells were stimulated with TNF-α (20 ng/mL) for 24 h in the presence or absence of calcitriol (100 nM). Cell lysates were immunoprecipitated with anti-p65 antibodies and analyzed by Western blotting using anti-VDR and anti-p65 antibodies. Total cell lysates were also immunoblotted with anti-VDR and anti-GAPDH antibodies. Representative images (**A**) and quantitative data (**B**) from five experiments (*n* = 5) were presented here. (**C**–**H**) Calcitriol-mediated anti-inflammatory response was partly blunted by *VDR* gene silencing in AC16 cells exposed to TNF-α. AC16 cells were transfected with si-NC or si-*VDR* and then stimulated with TNF-α (20 ng/mL) for 24 h in the presence or absence of calcitriol (100 nM). The representative Western blot images (**C**) and quantitative data (**D**) of VDR, IL-1β, and GAPDH in total cell lysates, and p-p65 and H3 in nuclear lysates from five experiments (*n* = 5) were presented here. (**E**–**H**) qRT-PCR analysis of *VDR, IL-1β, TNF-α,* and *IL-6* gene expression in AC16 cells (*n* = 5). (Data represent means ± S.D. * *p* < 0.05, ** *p* < 0.01, ^ns^ *p* > 0.05. One-way ANOVA with a post hoc test of Tukey’s analysis).

Our data have so far suggested that calcitriol prevents TNF-α-induced NF-κB p-p65 nuclear translocation and inhibits inflammation mainly through the VDR. To confirm such a notion, we first carried out a co-IP experiment with a p65 antibody and observed a much higher interaction between p65 and VDR in AC16 cells stimulated with both TNF-α and calcitriol (Figure 6A,B), suggesting that calcitriol promotes VDR binding to p65 in TNF-α-treated AC16 cells. We then conducted VDR knockdown experiments to study the functional importance of VDR signaling in our system. We found that all three *VDR*-gene specific siRNAs could successfully down-regulate VDR protein expression in AC16 cells, and si-*VDR*2 exhibited a better gene knockdown efficiency (Figure S6). This siRNA, therefore, was used in other knockdown experiments. The data shown in Figure 6C,D reveal that both TNF-α-induced IL-1β up-regulation and p65 nuclear translocation were significantly reduced by calcitriol, while such inhibitory effects were partially abolished by silencing the *VDR* gene. Similar effects were observed with other pro-inflammatory genes (*IL-1β, TNF-α, IL-6*) as detected by qRT-PCR (Figure 6E–H). These data demonstrate that VDR signaling is required for calcitriol-mediated anti-inflammatory response in cardiomyocytes.

### 3.8. Calcitriol Transcriptionally Upregulates IL-10 Expression by Promoting Direct Binding of VDR with the IL-10 Gene Promoter

**Figure 7:**
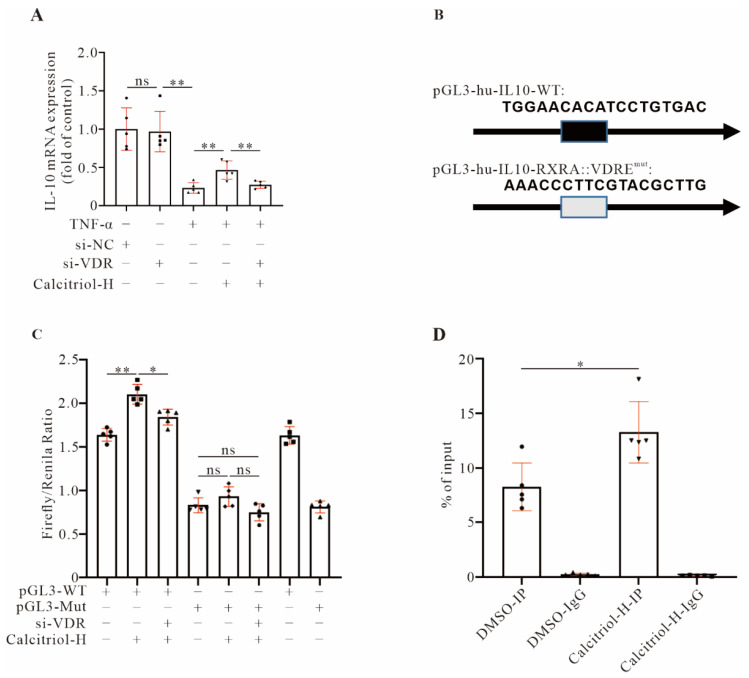
Calcitriol upregulated IL-10 expression by promoting direct binding of VDR with IL-10 gene promoter. (**A**) qRT-PCR analysis showed *IL-10* expression in AC16 cells with the indicated treatments. (**B**) Schematic illustration of human *IL-10* gene promoter vector harbouring a wild-type (pGL3-hu-IL-10-WT) or mutated version (pGL3-hu-IL-10-RXRA::VDRE^mut^) of RXRA/VDR response element. (**C**) Luciferase reporter assay was performed in AC16 cells with indicated treatments (*n* = 5). (**D**) CHIP assay showed calcitriol-H treatment promoted VDR binding to the human *IL-10* gene promoter (*n* = 5). (Data represent means ± S.D. * *p* < 0.05, ** *p* < 0.01, ^ns^ *p* > 0.05. Two- and three-way ANOVA with a post hoc test of Tukey’s analysis was used in (**D**) and (**A**)/(**C**), respectively).

IL-10, an anti-inflammatory cytokine, plays a protective role in the heart by preventing adverse cardiac remodeling [16,17]. We observed that IL-10 was upregulated by calcitriol in TNF-α treated cardiomyocytes and MI mice. Additionally, *IL-10* gene expression was significantly induced by calcitriol in TNF-α-treated cells, while such induction was completely abrogated by *VDR* gene knockdown (Figure 7A), supporting a critical role for the VDR in calcitriol-mediated *IL-10* gene expression. Interestingly, by scrutinizing the human *IL-10* gene promoter region, we found there was an RXRA::VDR binding element within the human *IL-10* gene promoter (Figure 7B). Luciferase assays showed that *IL-10* gene promoter activity was significantly increased by calcitriol, while such an increase was partially blunted when the *VDR* gene was knockdown (first three bars, Figure 7C). Importantly, no such modulation was observed when the RXRA::VDR binding element within the human *IL-10* gene promoter was mutated (4th to 6th bar, Figure 7C). Interestingly, we also observed decreased luciferase activity with a mutated vector without any additional treatment (last bar, Figure 7C), indicating that the RXRA::VDR binding element within the human *IL-10* gene promoter is important for basal *IL-10* gene transcription. Finally, ChIP-qPCR assay data showed that VDR could directly bind to the *IL-10* gene promoter, and such binding was further increased by calcitriol (Figure 7D). These data demonstrate that *IL-10* gene expression is upregulated by calcitriol at a transcriptional level, and the RXRA::VDR binding element within the human *IL-10* gene promoter is critical for *IL-10* gene transcription regulated by calcitriol.

## 4. Discussion

In this study, we have provided comprehensive evidence to confirm that supplementation of the active form of vitamin D3 could prevent MI-induced adverse cardiac remodeling, and even halt/reverse chronic MI development/progression. Mechanistically, we demonstrate that calcitriol exerts its cardioprotective effects by inhibiting cardiac inflammation and preventing cardiomyocyte death, thereby reversing MI-induced adverse cardiac remodeling. Furthermore, we show that calcitriol prevents MI-induced cardiac inflammation through two mechanisms: dampening NF-κB signaling via preventing p-p65 nuclear translocation and up-regulating *IL-10* gene expression.

It is well-documented that chronic deficiency of vitamin D is a detrimental condition for the cardiovascular system and represents an independent risk factor for multiple cardiovascular diseases including AMI and heart failure (HF) [18,19]. In this study, we observed a decrease in serum 25-OH vitamin D3 level in MI mice at 4 weeks post-surgery. This finding is perfectly consistent with the clinical data that a significantly low level of circulating 25-OH vitamin D3 is normally reported in patients with MI and/or HF. The underlying cause for such reduction is yet unclear, but we speculated that cardiovascular injury observed in MI mice or patients with MI and/or HF could impair multiple pathways of vitamin D production and metabolism including dietary intake, gut dysfunction, and vitamin D conversion to 25-OHD in the liver and other tissues, thereby reducing circulating 25-OH vitamin D3 levels.

The histology and echocardiography studies showed that calcitriol exhibited an apparent beneficial effect in preventing the progression of myocardial hypertrophy in post-MI mice, as indicated by decreased HW/BW ratio and ventricular cardiomyocyte size. Similarly, MI-induced upregulation of both NT-proBNP (the HF marker) and cTnI (myocardial injury marker) was significantly blunted by calcitriol, further supporting its protective properties against chronic MI development/progression. Previous research revealed that paricalcitol, an activated vitamin D2 analog, could reduce ventricular hypertrophy, improve cardiac function, and delay the progression of HF in transverse aortic constriction mice [20]. Moreover, anti-HF and anti-hypertrophic effects of paricalcitol have been reported in dietary sodium-induced HF in Dahl salt-sensitive animals [21,22]. Similar to these previous findings, we demonstrate that calcitriol supplementation can significantly prevent myocardial hypertrophy and improve cardiac function in mice after MI. Crucially, using a therapeutic protocol, we also demonstrate the therapeutic potential of calcitriol supplementation in pre-existing chronic MI.

Because the regenerative capacity of the adult mammalian heart is minimal, massive cardiomyocyte necrosis triggers an inflammation-driven reparative fibrotic response that leads to scarring [23]. It is characterized by increased deposition of type I collagen, and activation and differentiation of cardiac fibroblasts into myofibroblasts [24]. As the scar matures, fibroblasts in the infarct area lose their myofibroblast properties and may undergo apoptosis [25]. However, in larger myocardial infarctions, pressure and volume overload due to massive loss of contractile cells may lead to progressive interstitial fibrosis in the junctional zone and remote remodeling of the myocardium [26]. Our histological analysis revealed that calcitriol could attenuate interstitial and perivascular fibrosis in mice on both prevention and therapeutic protocols, suggesting an anti-fibrotic effect for calcitriol. Our observations are in line with previous findings, namely that synthetic vitamin D analogues, such as calcitriol and paricalcitol, are capable of reducing myocardial fibrosis and cardiac remodeling [11,20]. Particularly, one important finding in the current study is that we demonstrate that Calcitriol treatment started after 4 weeks from the MI is able to reduce the infarct area and improve cardiac functions. Considering that it was traditionally thought that the myocardial scare following MI is an irreversible process, our finding could provide an alternative view about this concept. The beneficial effects of late Calcitriol treatment on cardiac functions and remodeling could attribute to (1) Calcitriol protects cardiomyocytes from MI- or TNF-α-induced apoptosis and death, and also promotes cardiomyocytes proliferation, indicating that calcitriol may play a functional role in cardiac regeneration after MI by preserving or increasing pre-existing cardiomyocytes; (2) Calcitriol inhibits inflammation and reverse the harsh inflammatory environment in cardiac tissue after MI, further facilitating cardiac regeneration; (3) Calcitriol promotes angiogenesis which in turn reverses the adverse cardiac remodeling processes; (4) Calcitriol modulates the activation of some matrix metalloproteinases which in turn breaks down the fibrous matrix and remodels the myocardial scare; and (5) Calcitriol mobilizes and activates endogenous stem and progenitor cells which actively participate in heart regeneration. Therefore, it would be interesting to investigate which one or more abovementioned pathways are responsible for the beneficial effects of late Calcitriol treatment on cardiac functions and remodeling in our future studies.

Another interesting finding in the current study is that we show that reversing pathological aortic remodeling and restoring vascular reactivity are two beneficial effects of calcitriol on MI, among its other cardioprotective properties. Multiple cellular and molecular mechanisms could account for the beneficial effects on the vascular reactivity of treatment with Calcitriol. First, it has been well-documented that Vitamin D promotes vascular regeneration by increasing the number of angiogenic myeloid cells and promoting vascular reendothelialization after injury [27]. Second, similar to cardiac inflammation Vitamin D treatment also suppresses vascular inflammation, which in turn increases vascular reactivity. Third, Vitamin D treatment restores MI-induced vascular dysfunction may through its nongenomic functions including rapid generation of second messengers, quick opening of calcium channels, and activation of protein kinases [28].

Both innate immunity and inflammation are critical for cardiac tissue recovery from MI, via orchestrating beneficial cardiac repairing processes and maintaining homeostatic status in the infarcted heart. However, inappropriate and excessive inflammatory response leads to deterioration of cardiac function and irreversible cardiac remodeling, finally HF. After MI, the vulnerable myocardium containing necrotic tissues and inflammatory cells is easily affected by wall stress, leading to infarction enlargement [29,30]. Chronic inflammatory reactions occurred in the infarcted myocardium and excessive production of pro-inflammatory cytokines causes further injuries to the vulnerable myocardium and hinder the healing process of the infarcted heart [31]. Our previous study showed that Xin-Ji-Er-Kang, a Chinese herbal formula with cardioprotective properties, could inhibit cardiac inflammation and attenuate the progression of HF in mice post-MI [32]. Similarly, in the current study, we observed increased levels of pro-inflammatory cytokines (TNF-α and IL-1β), but a decreased level of anti-inflammatory cytokine IL-10 in the serum of MI mice. Importantly, such alterations could be normalized by calcitriol. NF-κB signaling plays a critical role in various physiological and pathological cellular events of cardiovascular disease [33,34]. We found that calcitriol mitigated inflammatory factor expression by inhibiting the NF-κB signaling pathway in TNF-α-treated AC16 cells and post-MI mice. Mechanistically, multiple biochemical assays including Western blot, immunofluorescence, and imaging flow cytometry analysis showed that MI- and TNF-α-induced NF-κB p-p65 nuclear translocation was prevented by calcitriol, indicating that calcitriol exerts its anti-inflammatory effects by dampening NF-κB signaling in the context of MI.

A normal physiological concentration of vitamin D is essential for the broad biological functions of vitamin D and its critical role in maintaining homeostasis [35]. Our data showed that calcitriol could increase both cytoplasmic and nuclear 1,25(OH)_2_-VitD_3_ levels in TNF-α-treated AC16 cells. As expected, calcitriol also induces increased expression of the VDR in infarcted myocardium and TNF-α-treated cardiomyocytes. Importantly, our data showed that the VDR could directly interact with p65 protein, and subsequently inhibits inflammation, indicating that calcitriol inhibits inflammation through the VDR. Such a notion is firmly supported by *VDR* gene knockdown data. Specifically, both inhibitory effects of calcitriol on pro-inflammatory cytokines expressions and NF-κB p-p65 nuclear translocation were lost when *VDR* gene expression was silenced.

Growing evidence has indicated that IL-10 plays a protective role in the damaging heart by mitigating adverse cardiac remodeling in response to pressure overload and angiotensin II stimulation [16,17,36] and that IL-10 activation could become a potential therapeutic in controlling pathological cardiac hypertrophy and preventing HF. Previous reports showed that in *IL-10* KO mice VDR, expression is required for the T cells and other immune cells to control gastrointestinal immunity and that vitamin D3 attenuates doxorubicin-induced cardiovascular toxicity through upregulation of IL-10 [37,38], suggesting a regulatory role for the VDR in *IL-10* gene expression. In this study, we provided multiple lines of evidence to confirm that VDR transcriptionally regulates *IL-10* gene expression. First, we observed that the *IL-10* gene was positively controlled in our system by the VDR, since silencing the *VDR* downregulated *IL-10* gene expression. Second, we found that *IL-10* gene promoter activity was closely regulated by the VDR, and the RXRA::VDR DNA element within the *IL-10* gene promoter is critical for such regulation. Third, CHIP assays confirmed a direct interaction between the VDR and *IL-10* gene promoters.

Our study has several limitations. First, it has been well documented that by binding and activating VDR signaling vitamin D also activates several other intracellular second messengers including PI3K/Akt and MAPK (ERK1/ERK2) [39], thereby exerting its anti-inflammatory response, which has not been explored in the current study. Second, since different batches of animals and experimental conditions/environments were used in different animal experiments due to the pandemic, mice included in Figure 3 displayed a less severe phenotype. Third, TNF-α doses used in cell-based studies were chosen based on previous studies ([13,14]), which are significantly higher than reported endogenous pathological levels of this cytokine in circulation. Last, additional evaluation of cell death and apoptosis using other methods such as caspase-3 cleavage and DNA laddering could be important to further support the observed inhibitory effect of calcitriol on cardiomyocyte apoptosis and death. Nevertheless, we have demonstrated both preventive and therapeutic potential for calcitriol in treating acute and established chronic MI in this study. Our study shows that the beneficial effects of calcitriol are partially attributed to its two anti-inflammatory actions: Calcitriol upregulates and activates VDR signaling, which in turn dampens the NF-κB pathway by interacting and preventing NF-κB p65 nuclear translocation. Meanwhile, activated/upregulated VDR increases *IL-10* gene transcriptional expression by direct binding to the *IL-10* gene promoter via RXRA::VDR element. Taken together, our study provides novel insights into the cardioprotective effects of calcitriol, and paves the way for its clinical treatment in acute and established chronic MI. However, it is worth mentioning that the cross-talk among the deficit or impaired action of vitamin D, inflammation, and insulin resistance plays a pivotal role in the pathogenesis of cardiovascular diseases, as exemplified by insulin resistance can be the consequence or the cause of deficiency/resistance of vitamin D, and many beneficial effects evoked by the vitamin D supplementation on the cardiovascular system are mediated by the improvement of insulin sensitivity ([40]). Therefore, insulin resistance correction could be necessary in order to optimize the beneficial effects of vitamin D supplementation in cardiovascular prevention.

## Data Availability

Not applicable.

## Supplementary Materials

The following supporting information can be downloaded at: https://www.mdpi.com/article/10.3390/cells11101676/s1, Figure S1: ECG and TTC staining in post-MI mice; Figure S2: Calcitriol reverses MI-induced adverse aortic remodeling and restores vascular reactivity; Figure S3: Immunofluorescence staining analysis of cardiac VDR expression in MI mice; Figure S4: Calcitriol prevented TNF-α induced AC16 cell death; Figure S5: Immunofluorescence staining analysis of VDR expression in AC16 cells; Figure S6: *VDR*-gene specific siRNA knockdown efficiency; Table S1: Sequences of oligonucleotides used as primers.

## Abbreviations

AR	aorta radius;
BNP	brain natriuretic peptide;
Co-IP	coimmunoprecipitation;
CSA	cross-sectional area;
cTnI	cardiac troponin I;
CVF	collagen volume fraction;
EF	ejection fraction;
FITC	fluorescein isothiocyanate;
FS	fractional shortening;
HE	hematoxylin and eosin;
HF	heart failure;
HW/BW	heart weight/body weight;
IL-1β	interleukin-1β;
IL-10	interleukin-10;
LAD	left anterior descending;
LUW/BW	lung weight/body weight;
LVAWd	left ventricular anterior wall thickness at diastole;
LVAWs	left ventricular anterior wall thickness at systole;
LVIDd	left ventricular internal diameters at diastole;
LVIDs	left ventricular internal diameters at systole;
MI	myocardial infarction;
NF-κB	nuclear factor kappa B;
NT-proBNP	N terminal pro B type natriuretic peptide;
PVCA	perivascular collagen area;
PVDF	polyvinylidene fluoride;
siRNA	small interfering RNA;
TAA	total aorta area;
TNF-α	tumor necrosis factor-α;
TTC	2,3,5-triphenyltetrazolium chloride;
VDR	vitamin D receptor.
