# Supplementary material for: Novel Insights into the Cardioprotective Effects of Calcitriol in Myocardial Infarction
Source: Cells. 2022 May 18;11(10):1676. doi: 10.3390/cells11101676 (PMC9139780; doi:10.3390/cells11101676)
Supplement: Supplementary file 1 [file cells-11-01676-s001.zip › cells-1665902-supplementary.pdf]

Figure S1: ECG and TTC staining in post-MI mice. (A) Representative ECG charts of five mice with the indicated treatments. ECG measurements were conducted at five minutes before (I) and after surgery (II), as well as four weeks post-MI (III), respectively. (B) ECG parameters at four weeks post-MI. (C) representative images showing TTC staining of myocardial infarct area at 24 hours after MI. Data presented here are representative images (A and C) or quantitative analysis (B) from five mice (n=5 mice/group), respectively. (Data represent means ± S.D. **P<0.01, versus sham group, #P<0.05, ##P<0.01 versus MI group; One-way ANOVA with a post hoc test of Tukey's analysis).

A

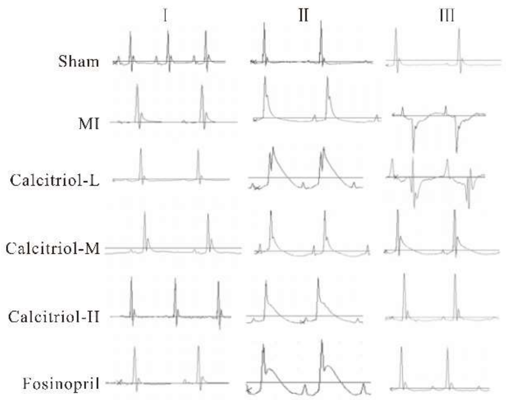

B

Group	Sham	MI	Calcitriol-L	Calcitriol-M	Calcitriol-H	Fosinopril
Height of P (mv)	0.06±0.01	0.14±0.01**	0.14±0.02	0.12±0.01$^{\#\#}$	0.10±0.02$^{\#\#}$	0.11±0.02$^{\#\#}$
Width of P (ms)	10.75±1.49	21.75±2.87**	17.86±2.19$^{\#}$	15.67±1.97$^{\#\#}$	14.00±1.29$^{\#\#}$	12.88±1.64$^{\#\#}$
Height of QRS (mv)	0.85±0.16	0.27±0.12**	0.35±0.15	0.41±0.08$^{\#}$	0.72±0.22$^{\#\#}$	0.79±0.09$^{\#\#}$
Width of QRS (ms)	14.50±1.60	21.63±2.83**	19.29±2.56	15.83+2.23$^{\#\#}$	14.14±1.68$^{\#\#}$	14.75±2.31$^{\#\#}$
Width of S (ms)	4.25±0.71	11.63±1.92**	11.86±1.07	8.83±1.47$^{\#\#}$	5.86±1.35$^{\#\#}$	5.63±0.92$^{\#\#}$
Height of J Point (mv)	0.00±0.00	0.14±0.06**	0.04±0.04$^{\#}$	0.05±0.03	0.01±0.01$^{\#\#}$	0.00±0.00$^{\#\#}$
Height of T (mv)	0.14±0.06	-0.11±0.05**	-0.07±0.04	-0.09±0.04	0.10±0.02$^{\#\#}$	0.11±0.02$^{\#\#}$
Width of T (ms)	10.50±2.39	18.63±1.69**	17.71±2.81	14.50±1.64$^{\#\#}$	10.57±1.13$^{\#\#}$	13.00±1.77$^{\#\#}$
PR interval (ms)	40.88±3.23	34.00±4.17**	31.14±1.86	39.50±6.92	41.71±4.61$^{\#\#}$	36.50±2.45
QT interval (ms)	24.13±3.18	40.25±3.62**	36.86±3.89	30.50±3.51$^{\#\#}$	24.57±1.90$^{\#\#}$	27.63±3.62$^{\#\#}$

C

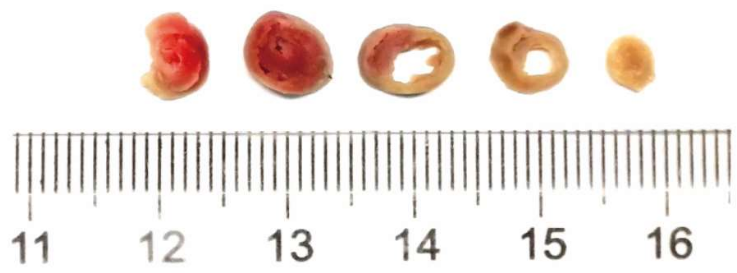

Figure S2: Calcitriol reverses MI-induced adverse aortic remodeling and restores vascular reactivity. (A) Representative images of aortic remodeling in different groups. (B-E) Quantitative analysis of total aortic wall area (TAA), aorta radius (AR), luminal radius (Lumen), and media thickness (Media) from eight mice (n=8 mice/group), Representative images (F) and quantitative analysis (G) of Masson staining of thoracic aorta from six mice (n=6 mice/group). (H-I) Vasodilatation in response to acetylcholine (Ach, H) and sodium nitroprusside (SNP, I), respectively (n=6 mice/group). Scale bar represents 100 μm. $^{*}P < 0.05$, $^{**}P < 0.01$ versus sham group, $^{\#}P < 0.05$, $^{\#\#}P < 0.01$ versus MI group (One- or two-way ANOVA with a post hoc test of Tukey's analysis).

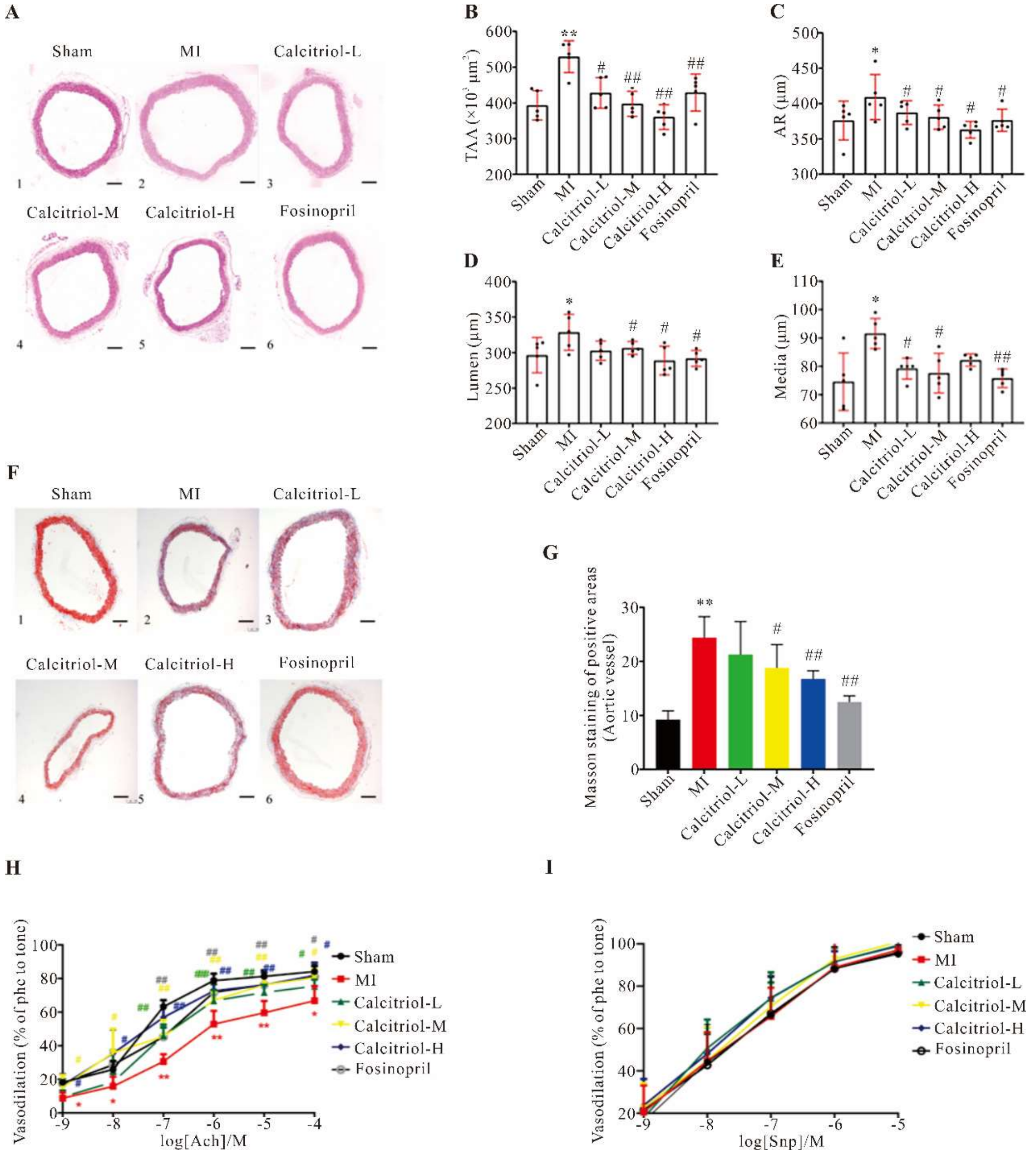

Figure S3: Immunofluorescence staining analysis of cardiac VDR expression in MI mice. Representative images (A) and quantification (B) of VDR expression in cardiac tissues from five mice (n=5 mice/group). Scale bar represents 50 μm. $^{**}P < 0.01$ versus sham group, $^{\#\#}P < 0.01$ versus MI group (One-way ANOVA with a post hoc test of Tukey's analysis).

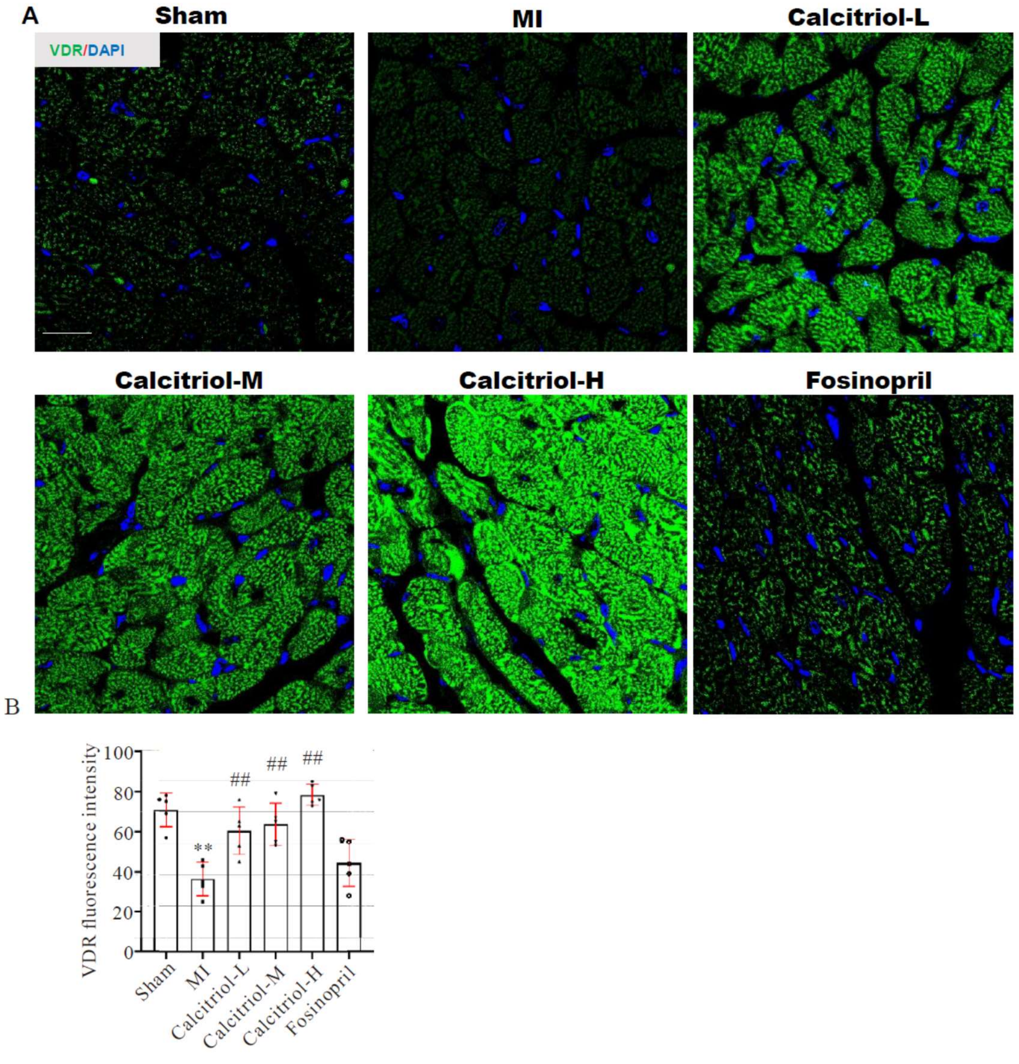

Figure S4: Calcitriol prevented TNF-α induced AC16 cell death. (A) The effect of TNF-α at different doses on cell viability in AC16 cells (n=5). (B) The effect of TNF-α (20 ng/ml) on cell viability at different times in AC16 cells (n=5). (C) The influence of TNF-α, DMSO, calcitriol-L and calcitriol-H on cell viability (n=5). (D, E) Cell numbers and confluence in AC16 cells with the indicated treatments from 0 to 24 hours (n=5). (F, G) PCNA protein expression in AC16 cells with various treatments (n=5). (H) Immunofluorescence staining analysis of PCNA and ki67 in AC16 cells with the indicated treatments (n=5). (I-J) Immunofluorescence staining analysis of cleaved caspase-3 in AC16 cells with different treatments. Scale bars in H and I represent 50 μm and 10μm, respectively.$^{**}P < 0.01$ versus control group, $^{\#}P < 0.05$ versus TNF-α (20 ng/ml) group (One- or two-way ANOVA with a post hoc test of Tukey's analysis).

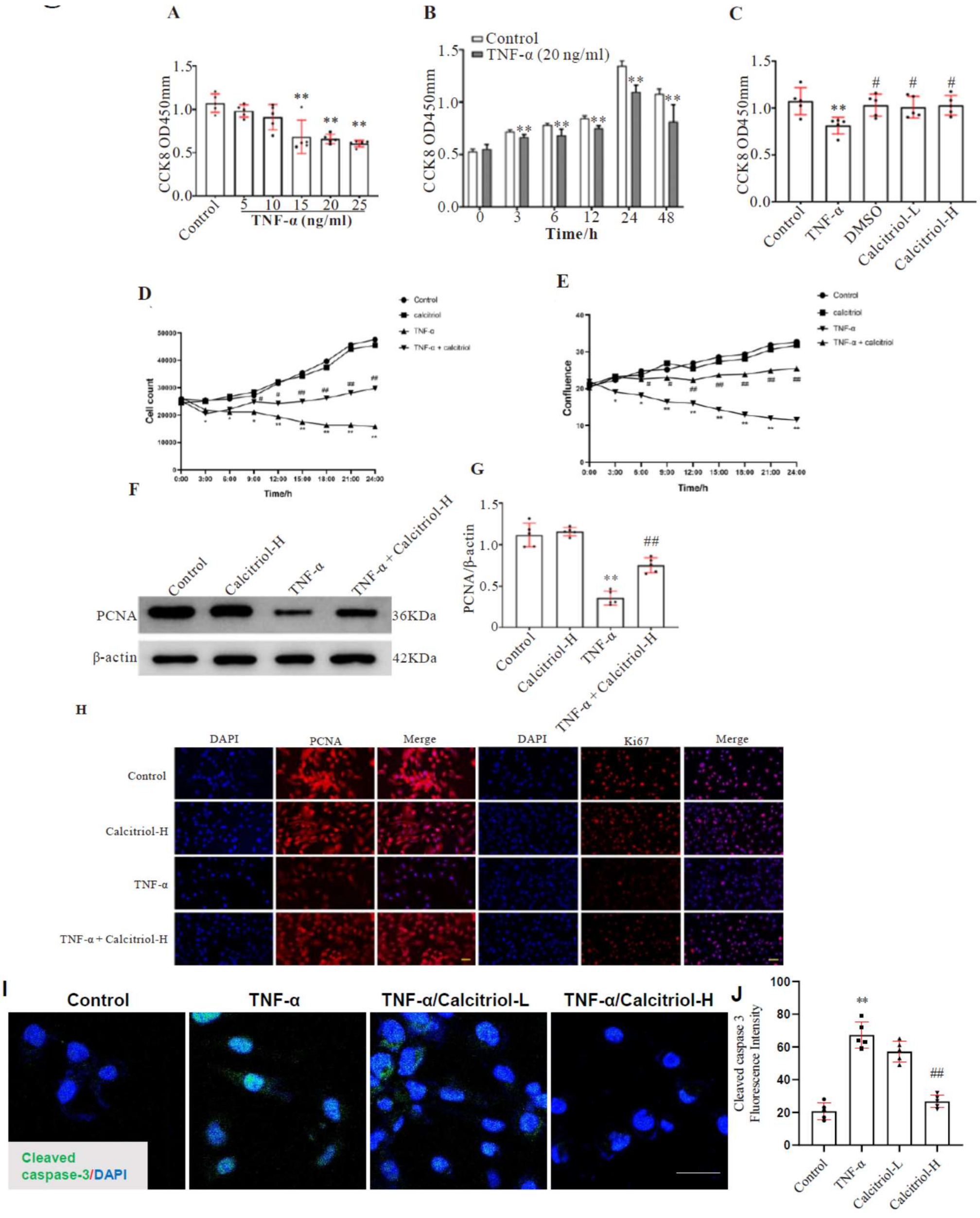

Figure S5: Immunofluorescence staining analysis of VDR expression in AC16 cells. (A) Representative immunofluorescence microscopic images showing VDR expression in AC16 cells. (B) The quantification of fluorescence intensity of VDR expression in AC16 cells (n=5). Scale bar represents 50 μm. $^{**}P < 0.01$ versus sham group, $^{\#\#}P < 0.01$ versus MI group ((One-way ANOVA with a post hoc test of Tukey's analysis)).

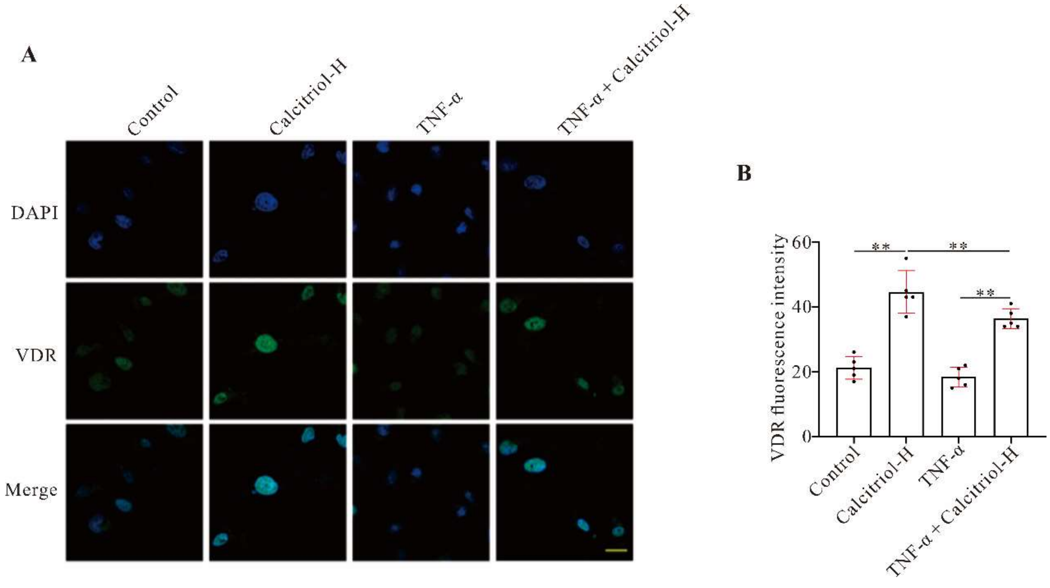

Figure S6: VDR-gene specific siRNA knockdown efficiency. Representative images of VDR expression of three different products and quantitative analysis by Western blotting (n=5). $^{**}P < 0.01$ versus control group.

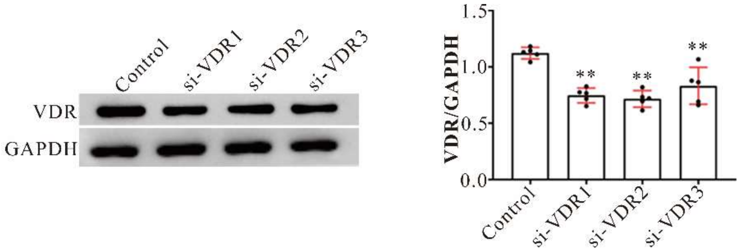

Supplemental Table S1. Sequences of oligonucleotides used as primers.

Target gene		Sequence (5'-3')
VDR	Forward primer	CCACTGGCTTTCACTTCA
	Reverse primer	TCATCTCCCGCTTCCTCT
IL-β	Forward primer	ACAGTGGCAATGAGGATG
	Reverse primer	TGTAGTGGTGGTCGGAGA
IL-6	Forward primer	GTCCAGTTGCCTTCTCCC
	Reverse primer	GCCTCTTTGCTGCTTTCA
TNF-α	Forward primer	CGAGTGACAAGCCTGTAGCC
	Reverse primer	TGAAGAGGACCTGGGAGTAGAT
IL-10	Forward primer	ACCAAGACCCAGACATCA
	Reverse primer	TTCACAGGGAAGAAATCG
β-actin	Forward primer	CATGTACGTTGCTATCCAGGC
	Reverse primer	CTCCTTAATGTCACGCACGAT
